# *Oenanthe javanica* Ethanolic Extract Alleviates Inflammation and Modifies Gut Microbiota in Mice with DSS-Induced Colitis

**DOI:** 10.3390/antiox11122429

**Published:** 2022-12-09

**Authors:** Ui-Jin Bae, Ha-Na Jang, Sung-Hyen Lee, Ji-Young Kim, Gi-Chang Kim

**Affiliations:** National Institute of Agricultural Sciences, Wanju 55365, Republic of Korea

**Keywords:** colitis, inflammation, oxidative stress, microbiota, *Oenanthe javanica*, dextran sulfate sodium

## Abstract

*Oenanthe javanica*, commonly known as water dropwort, has long been used to treat acute and chronic hepatitis, abdominal pain, alcohol hangovers, and inflammation in various traditional medicine systems in Asia. However, whether *O. javanica* has beneficial effects on colitis-induced intestinal damage remains elusive. This study tested the hypothesis that *O. javanica* has anti-inflammatory and antioxidant activities in mice with dextran sulfate sodium (DSS)-induced colitis. First, treatment of *O. javanica* ethanol extract (OJE) inhibited the production of inflammatory cytokines in lipopolysaccharide-affected macrophages. Second, in mice with DSS-induced colitis, OJE administration reduced pathological damage to the colon while alleviating weight gain and decreasing colon length, including inflammation and mucosal necrosis. In addition, OJE significantly (*p* < 0.01) restricted the activation of nuclear factor-κB (NF-κB) and the secretion of pro-inflammatory mediators and increased the expression of Nrf2-phase 2 antioxidant enzymes. The results of 16S rRNA gene sequencing workflows for taxonomic assignment analysis confirmed that the diversity (richness and evenness) of fecal microbiota was markedly elevated in the OJE group. OJE administration reduced the abundance of *Proteobacteria* including *Escherichia* and increased the abundance of the genus *Muribaculum*. These results suggested that OJE exerts beneficial effects on inflammation and gut microbial composition in a mouse model of colitis.

## 1. Introduction

Inflammatory bowel disease (IBD) is a chronic, recurrent inflammatory disease of the intestine that primarily comprises Crohn’s disease and ulcerative colitis (UC) [1]. The incidence of IBD is gradually increasing in Western countries and Asia, including Republic of Korea [2]. These symptoms result from inflammation-mediated destruction of the surface of the mucosal layer in the gastrointestinal tract [3]. UC is an immune system disorder that destroys the intestinal mucosa and triggers an inflammatory response. It has been reported that environmental factors such as smoking and stress, genetic factors, and intestinal bacteria act in a complex way [4]. Currently, clinical treatments for UC include anti-inflammatory drugs, such as sulfasalazine, 5-aminosalicylic acid (5-ASA), corticosteroids, monoclonal antibodies, and immune suppressants [5]; however, these are not permanent drugs for the complete cure of colitis. Despite understanding the numerous causes of UC, applicable medical treatments are limited. Most of these drugs have only temporary effects, such as relieving diarrhea or pain, and serious adverse reactions such as nausea, abdominal pain, kidney damage, and hepatotoxicity, when used in high doses or for a long time [6]. Therefore, it is advantageous to identify phytochemicals derived from edible plants that are safe for the general public to minimize the side effects of IBD and for effective treatment. As most functional phytochemicals have fewer side effects than conventional drugs, they have recently attracted attention in disease treatment, and medicinal plants contribute to alleviating and improving symptoms in patients with IBD [7].

Most phytochemicals are reported to inhibit inflammation by directly or indirectly regulating the activation of NF-κB [8]. In addition, various functions are known, including the activity relationship owing to the structure of phytochemicals and the starting point for leading drug optimization studies [9]. To understand and effectively treat IBD, various genetic, immunological factors, and environmental factors must be considered. For example, while the gut microbiome is important, even in healthy individuals, it plays a particularly important role in many aspects of IBD [10]. The amount of food supplied, and the composition of nutrients have been shown to be two of the most important factors influencing the gut microbiota [11]. Consequently, diet may be a viable option for the treatment and prevention of IBD. However, studies on the effects of ingestion of each nutrient on the management and prevention of diseases are lacking and require in-depth consideration.

*Oenanthe javanica* (Blume) DC. (Apiaceae), a small perennial herb, has been cultivated in tropical and temperate Asia for thousands of years and has been used as a folk remedy for various diseases. Various bioactivities of *O. javanica* have hepatoprotection [12,13], anti-inflammatory [14,15], ethanol elimination [16], immune-enhancement [17], anti-oxidant [18], and antiviral effects [19]. In other words, *O. javanica* is a valuable herbal plant that is used for food and various medicinal purposes in East Asian countries. For example, its flowers and stems are commonly used for the treatment of various types of acute and chronic hepatitis, jaundice, fever, hypertension, abdominal pain, and urinary tract issues, fever and high blood pressure in oriental medicine [20,21,22,23,24,25]. This plant has also been widely used in salads and soups as a dietary product, and as a folk remedy for alcohol hangovers and inflammatory conditions [26]. Based on the results of the aforementioned studies, it is reasoned that an improvement effect can also be observed in the IBD inflammation model.

We hypothesized that phenolic compounds in *O*. *javanica* ethanol extract (OJE) have a positive effect on colitis symptoms and gut microbiota. Therefore, this study aimed to investigate the potential anti-inflammatory and antioxidant effects of OJE by in vitro and in a mouse model of colitis. Furthermore, we focus on the effects of OJE intake to host on changes in the gut microbiota, which is another index of colitis symptoms and is closely related to inflammatory response in IBD.

## 2. Materials and Methods

### 2.1. Preparation of OJE

Dried *O. javanica* was purchased from a farm (35°21′25″ N, 127°10′10″ E; Republic of Korea). *O. javanica* was washed with distilled water and dehydrated. Washed *O. javanica* was homogenized for 2 min with a homogenizer (T50 digital ULTRA-TURRAX, IKA, Staufen, Germany) in 70% ethanol and stored in the dark at 25 °C for 48 h. Thereafter, the extracted solution was filtered and lyophilized using a freeze dryer (IlShinBioBase Corporation, Dongducheon, Republic of Korea). The dry weight of the extract was 46.26 g (yield, 10.8%).

### 2.2. LC/MS Analysis and Identification

Individual phenolic acid and flavonoid derivatives were analyzed using a liquid chromatography/mass spectrometer (LC/MS) (Sciex Co., Framingham, MA, USA) equipped with a CORTECS UPLC T3 column (150 × 2.1 mm i.d., 1.6 μm, Waters, Wexford, Ireland). The detailed conditions were as follows: flow rate (0.3 mL/min), column oven temperature (30 °C), and representative wavelength (phenolic acid 320 nm; flavonoid 350 nm). The mobile phase consisted of water (A) and acetonitrile (B), both of which contained 0.5% formic acid. The chromatographic gradient condition was: initial 5% B; 20 min, 25% B; 25 min, 50% B; 30 min, 90% B; 32 min, 90% B; 35 min, 5% B; and 40 min, 5% B. The mass spectrometric conditions were as follows: ion source gas 50 psi, curtain gas 30 psi, ion source temperature 500 °C, declustering potential 80 V, collision energy 15 V, spray voltage 5500 V, CE spread 10 V, and mass range (*m*/*z* 100–2000) in positive ionization mode.

The phenolic acid and flavonoid libraries of *Ruta graveolens, Carpobrotus edulis* ([27,28]: rutin 1), *Hibiscus sabdariffa* (http://koreanfood.rda.go.kr; RND DB 2.0 Phenolic Acids: rutin 2, last modified 30 September 2022), *Zizyphus jujuba* var. *inermis* (Bunge) Rehder ([29,30,31]: rutin 3) were constructed using data from the literature. Phenolic acid and flavonoid derivatives were identified from the data of these libraries, comprising compound names, used parts, molecular weights, and MS fragment ion patterns. Based on nine standards confirmed and selected by multiple reaction monitoring (MRM) of OJ (*O. javanica*), two phenolic acids and one flavonoid derivative were identified.

### 2.3. Cell Culture and Viability Assay

RAW 264.7, a macrophage cell line, was purchased from the American Type Culture Collection (ATCC, Rockville, MD, USA). Cells were maintained at 37 °C in a humidified 5% CO_2_ atmosphere in DMEM (Thermo Fisher Scientific, Warsaw, Poland) with 10% (*v*/*v*) fetal bovine serum (FBS; Gibco, Gaithersburg, MD, USA) and 1% penicillin/streptomycin (Thermo Fisher Scientific, Warsaw, Poland) composition. The cytotoxicity on RAW 264.7 macrophages was determined using the 2,5-diphenyl-2H-tetrazolium bromide (MTT) assay. Briefly, cells were seeded in 96-well plates (1 × 10^3^ cells/well) and incubated for 1 d, then incubated with various concentrations of OJE (5, 10, 20, or 40 μg/mL) for 24 h. To confirm cell viability, DMEM was changed to MTT solution (50 μL of 5 mg/mL), and incubated for 1 h. Following removal of the supernatant, each well was treated with 200 μL of dimethyl sulfoxide to dissolve the formed formazan. Absorbance was measured at 540 nm using a multimode microplate reader (SpectraMax M2, San Jose, CA, USA).

### 2.4. Nitric Oxide (NO) Production

RAW 264.7 cells were cultured in 96-well plates in DMEM at 37 °C in a 5% CO_2_ incubator for 24 h. After 4 h of pretreatment with OJE at different concentrations (5, 10, 20, or 40 μg/mL) cells were treated with lipopolysaccharide (LPS; 10 ng/mL) and cultured for 24 h. Thereafter, the culture supernatant was mixed with Griess reagent (Promega Corporation, Madison, WI, USA) (1:1, *v*:*v*) and reacted after blocking sunlight for 10 min at 25 °C, and the absorbance was measured at 540 nm.

### 2.5. Animal Study Design (Induction of Colitis and Treatment)

Sixty male BALB/c 6-week-old mice were purchased from Central Laboratory Animal, Inc. (Seoul, Republic of Korea) and housed in cages under standard conditions (22 ± 2 °C, 50–60% humidity, 12 h light-dark cycles) throughout the experiment. Mice were fed a standard laboratory chow diet ad libitum. The animals were randomly divided into six groups: group 1, control (CON, without colitis induction, *n* = 10); group 2, negative control (NC, DSS treatment, no OJE treatment, *n* = 10); group 3, positive control (5-ASA 50 mg/kg treatment with colitis induction, *n* = 10); group 4, OJE 50 mg/kg (with colitis induction, *n* = 10); group 5, OJE 100 mg/kg (with colitis induction, *n* = 10); group 6, OJE 200 mg/kg (with colitis induction, *n* = 10). After a 1-week adaptation period to the normal diet, colitis was induced by adding DSS (3%) to drinking water on days 0–14. For 14 d, groups 3–5 were administered oral doses of OJE daily and group 6 was orally administered 5-ASA daily. Body weight was monitored daily during DSS administration. The Institutional Review Board (IRB) guidelines for the ethical use of experimental animals were approved by the National Institute of Agricultural Sciences (approval no: NAS-202203) of Republic of Korea.

### 2.6. Histological Analysis and Colitis Scoring

On day 14, the mice were sacrificed, the entire colon collected, the tissue immediately washed with phosphate-buffered saline (pH = 7.4), and the length measured. As a staining preparation step for pathological reading of the tissue, the colon was fixed in 4% paraformaldehyde and cut to a size of 2–3 mm. Following deparaffinization and hydration, slides of approximately 3 μm were made using a microtome (Finesse ME Microtome, Thermo Shandon, city, state, country) and stained with hematoxylin and eosin (H&E). F4/80 as surface marker commonly used to label macrophages [32]. Immunostaining was performed using an antibody against F4/80 (Abcam, Cambridge, MA, USA) on the same slide. In this study, the findings were determined by referring to the International Harmonization of Nomenclature and Diagnostic Criteria, the necrosis and inflammation of the specimens were compared, and mucosal damage was assessed based on previously described criteria. The mucosal damage score was the sum of these three parameters for a maximum possible score of (10) [33]. To assess the severity of colitis, the Disease Activity Index (DAI) score for each group was determined based on the method of Friedman et al. [34], and the score was calculated as the sum of weight loss, diarrhea, and hematochezia scores.

### 2.7. Quantitative Real-Time Polymerase Chain Reaction (qRT-PCR)

Total RNA was isolated from colonic tissues (the rectum part 1~2 cm away from the anal canal, 0.3~0.5 × 0.3~0.5 cm) using TRIzol reagent (Invitrogen, Carlsbad, CA, USA). First-strand cDNA was generated using random hexamer primers, and the specific primers used for qPCR analysis for each gene are listed in Supplementary Table S1 (forward, FOR; reverse, REV). The qPCR reaction included 10 ng reverse-transcribed total RNA, 200 nM of each primer, and a PCR master mixture. Real-time qPCR was performed using an ABI QuantStudio6 Flex Real-time PCR System (Applied Biosystems, Foster City, CA, USA).

### 2.8. Western Blot Analysis

Total protein of colonic tissues and cells was extracted using a protein extraction reagent (Pierce Biotechnology, Rockford, IL, USA). Homogenates containing 20 μg of total protein were separated by SDS-PAGE and transferred to nitrocellulose membranes, and blocked with 5% bovine serum albumin in Tris-buffered saline containing 0.1% Tween 20. The membranes were probed with primary antibodies for 12 h, followed by the secondary antibodies horseradish peroxidase-conjugated IgG (Cell Signaling Technology, Beverly, MA, USA) for 1 h. Antibodies against iNOS (ab3523), COX-2 (ab15191), IL-1β (ab9722), TNF-α (ab9739), IκBα (ab32518), p-IκBα (ab133462), p-p65 (ab86299), Lamin B1 (ab16048), NQO1 (ab34173), HO-1 (ab13248), Nrf2 (ab92946) and β-actin (ab8227; Abcam, Cambridge, United Kingdom) were used. The membrane was incubated for 2 h at 25 °C after treatment with the secondary antibodies. After washing with Tris-buffered saline with Tween buffer, the membranes were treated with enhanced chemiluminescence solution and imaged using a Chem-iDoc system (Bio-Rad, Hercules, CA, USA). Imaged bands were quantified with β-actin using ImageJ software (Ver.1.8, National Institute of Health, Sacaton, Arizona, USA).

### 2.9. Enzyme-Linked Immunosorbent Assay

The collected blood samples from the inferior vena cava in mice were centrifuged at 2000× *g* for 20 min. The separated plasma was placed in a sterilized tube and stored at −60 °C for one week for use as an experimental sample. The supernatant was collected and the ELISA kit (R&D Systems, Minneapolis, MN, USA) was used to immediately quantify the active plasma levels of TNF-α, IL-6, IL-1β, and MCP-1 according to the manufacturer’s protocol. The absorbance of reacted samples was measured at 450 nm using a microplate reader.

### 2.10. 16S rRNA Gene Sequencing and Taxonomic Assignment

Bacterial DNA in mouse feces was extracted using a PowerSoil^®^ DNA isolation kit (#12888, Mo Bio, Carlsbad, CA, USA) on day 14th day after initiation of DSS treatment and addition of OJE. An amplicon of variable regions V3-V4 in 16S rRNA was amplified using universal primers (forward: 5′-TCGTCGGCAGCGTCAGATGTGTATAAGAGACAGCCTACGGGNGGCWGCAG-3′ and reverse: 5′-GTCTCGTGGGCTCGGAGATGTGTATAAGAGACAGGACTACHVGGTATCTAATCC-3′) with standard Illumina barcodes and adapters. Purified polymerase chain reaction products were normalized and pooled using PicoGreen, and NGS library size analysis was performed using TapeStation DNA ScreenTape D1000 (Agilent Tech., Santa Clara, CA, USA) followed by MiSeq™ Sequencing using a platform (Illumina, San Diego, CA, USA). Primers were trimmed using SeqPurge and the length of the short reads was adjusted by merging paired-end reads using FLASH (v1.2.11). The resulting 16S rRNA gene sequences were analyzed using the bioinformatics pipeline QIIME2 (ver. 2021.4). The demultiplexed raw sequence data were quality-filtered and denoised, and the amplicon sequence variants (ASVs) were obtained using the Divisive Amplicon Denoising Algorithm 2 (DADA2, https://benjjneb.github.io/dada2/, last modified 30 June 2022) algorithm as a QIIME plugin. Taxonomy was assigned using the SILVA v128 (universal approach) and SILVA v132 (archaeal approach) reference databases. A comparative analysis of the intestinal microbial community between the control and experimental groups was performed using ASV abundance and taxonomy information. To understand the richness and evenness of a single sample, alpha diversity was measured using the Shannon Index. Phylogenetic distances were measured to compare beta diversity among groups using weighted UniFrac principal coordinate analysis (PCoA) of the phyloseq R package. Linear discriminant effect size analysis (LEfse), based on the Galaxy web application method (http://huttenhower.sph.harvard.edu/galaxy/, last modified 30 June 2022) [35], was used to identify features that were most likely to explain group differences.

### 2.11. Statistical Analyses

Data are expressed as the mean ± standard error of the mean (SEM). Significant differences between the two groups were determined using the Student’s unpaired *t*-test. Statistical significance was set *p* < 0.05.

## 3. Results

### 3.1. LC/MS Analysis and Identification of OJE

**Figure 1:**
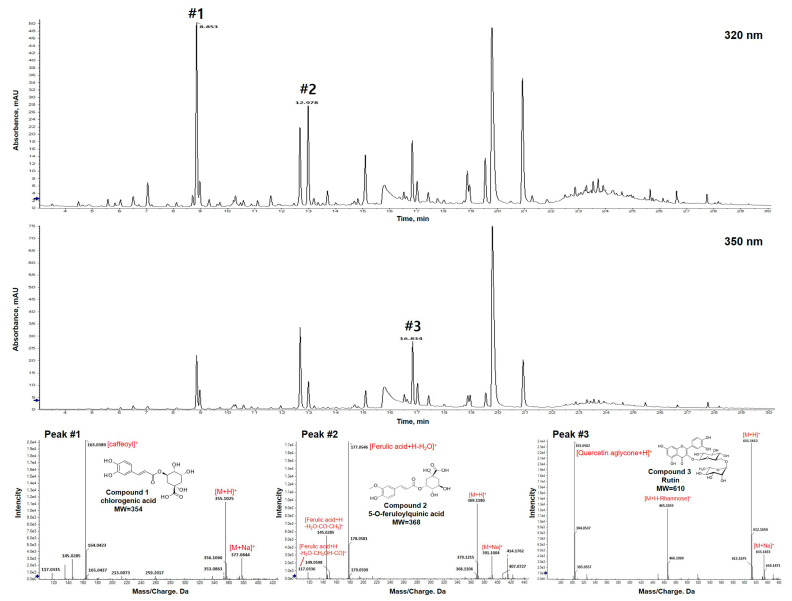
LC/MS analysis and identification of OJE. Phenolic acid and flavonoid derivative compounds in OJE. Numbers refer to the main peaks identified: #1, chlorogenic acid; #2, 5-O-feruloylquinic acid; #3, rutin (quercetin-3-rutinoside).

We obtained numerous chromatogram peaks from OJE using LC-MS and identified three compounds with mass fragment information, as reported in other studies (Supplementary Table S2). The mass fragments of peak 1 were 377, 355, and 169 *m*/*z*. Peak 1 was identified as chlorogenic acid with a molecular weight of 354 Da by confirming 377 *m*/*z* with Na^+^ binding to chlorogenic acid, 355 *m*/*z* with H + binding, and 169 *m*/*z* from [caffeoyl]^+^. For Peak 2, *m*/*z* 391 [M + Na]^+^, 369 [M + H]^+^, 177 [Fe^3^ + H-H_2_O]^+^, 145 [Fe + H-H_2_O-CO-CH_3_]^+^, and 117 Fe + H-H_2_O-CH_3_OH-CO]^+^ were identified and assumed to be 5-O-feruloyl quinic acid with a molecular weight of 368. Peak 3 was *m*/*z* 633 [M + Na]^+^, 611 [M + H]^+^, 465 [M + H-Rhamnose]^+^, 303 [quercetin aglycone + H]^+^, and putative quercetin-3-rutinosides of the rutin series. The main components of OJE are shown in Figure 1. Peaks #1, #2, and #3 were identified as chlorogenic acid, 5-*O*-feruloylquinic acid, and quercetin-3-rutinoside, respectively (Figure 1).

### 3.2. Anti-Inflammatory Effect of OJE on LPS-Stimulated RAW 264.7 Cells

**Figure 2:**
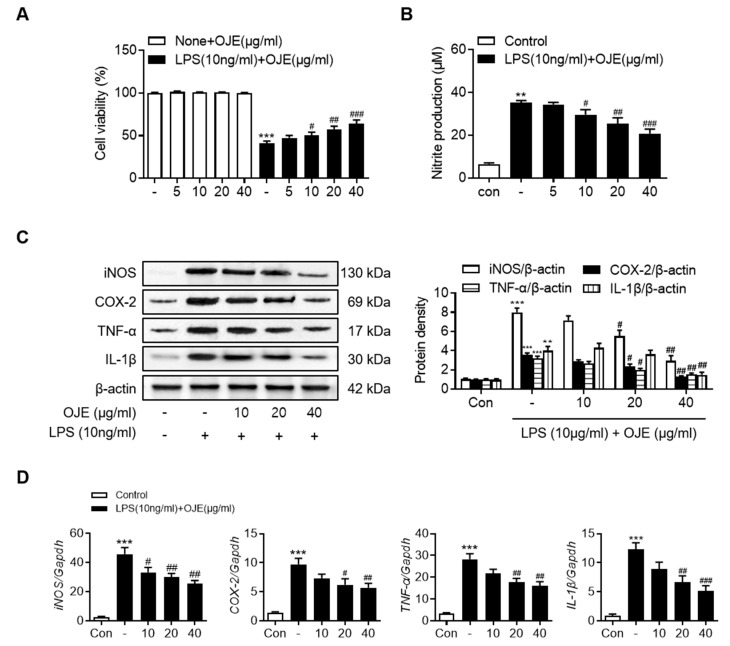
Effect of OJE on (**A**) cell viability (**B**) NO generation and mRNA and protein expression of iNOS, COX-2, TNF−α and IL-1β by (**C**) Western blotting, and (**D**) RT-qPCR in LPS-stimulated Raw 264.7 cells. The data are shown as the mean ±SD (*n* = 8). ** *p* < 0.01, *** *p* < 0.001 vs. normal (control); # *p* < 0.05, ## *p* < 0.01, ### *p* < 0.001 vs. LPS-stimulated Raw 264.7 cells without OJE.

The effect of the inflammatory response after addition of OJE obtained by 70% ethanol extraction on LPS-stimulated RAW 264.7 cells was investigated. Treatment with LPS significantly (*p* < 0.001) reduced cell viability to 59.12 ± 7.4% of that of control cells; however, pretreatment with OJE increased the viability of LPS-treated cells in a concentration-dependent manner (Figure 2A). Treatment with OJE alone had no effect on cell viability at the highest concentrations used in these experiments. In LPS-treated macrophages, the OJE-treated group showed a significant (*p* < 0.05) dose-dependent decrease in the production of the inflammatory mediator NO when compared with the NC group (Figure 2B). The experiment was performed in the same manner as described above, and the expression changes of iNOS and COX-2 in macrophages were confirmed by Western blotting. As shown in Figure 2C, OJE decreased the secretion of iNOS, COX-2, TNF-α, and IL-1β in inflammatory macrophages in a dose-dependent manner. In particular, the production of pro-inflammatory enzymes and cytokines significantly (*p* < 0.01) decreased in the OJE-40 administration group. The increase in the OJE concentration significantly (*p* < 0.05) decreased the mRNA levels of iNOS, COX-2, TNF-α, and IL-1β, similar to the Western blot results (Figure 2D).

### 3.3. OJE Supplementation Alleviates Colitis Symptoms in Mice with DSS-Induced Colitis

**Figure 3:**
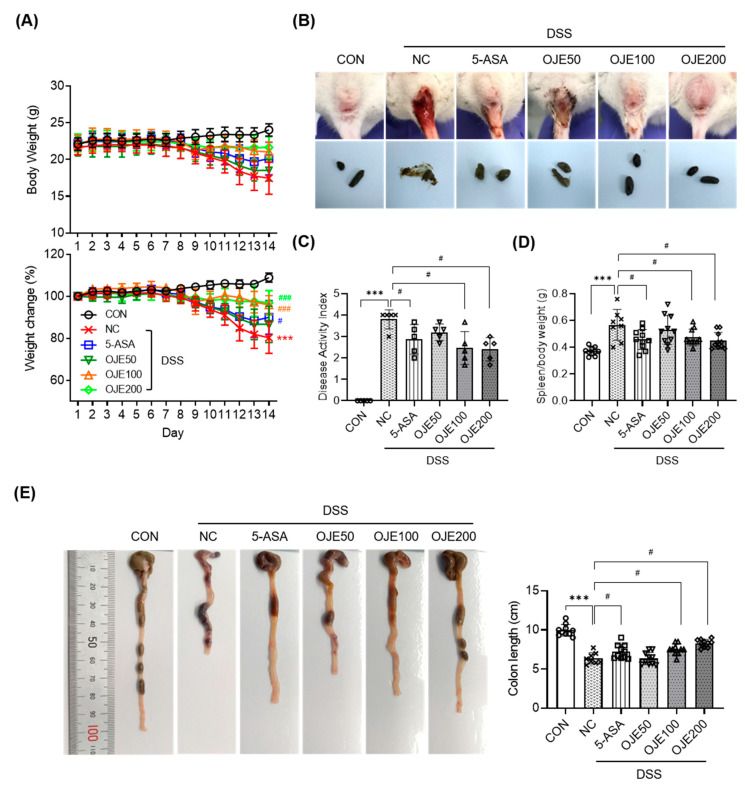
Effects of OJE administration on (**A**) body weight, weight change rate, (**B**) feces, anus and (**C**) DAI score (**D**) spleen/body weight and (**E**) colon length in mice with DSS-colitis. Values are the mean ± SEM (*n* = 8–10). *** *p* < 0.001, NC vs. CON; # *p* < 0.05, ### *p* < 0.001, 5-ASA 50 mg/kg, OJE 50 mg/kg, OJE 100 mg/kg, OJE 200 mg/kg vs. DSS+NC.

Similar to most studies [36], the DSS-induced mice used in this study also exhibited symptoms characteristic of colitis, and mice in the DSS group showed significant (*p* < 0.001) weight loss compared to those in the control group (Figure 3A). In particular, during the course of colitis in mice, weight loss, diarrhea, and severe bloody stools improved in the OJE (100 or 200 mg/kg) group (Figure 3A,B). The DAI score, the colitis assessment index, significantly (*p* < 0.05) increased in the presence of DSS; however, it decreased sharply in the presence of OJE (100 or 200 mg/kg) (Figure 3C). The spleen is a part of the lymphatic system in the body [37]. The lymphatic system helps to remove cellular waste, maintains fluid balance, and builds and activates infection-fighting white blood cells for the immune system. When a severe inflammatory response occurred, the spleen weight observed in the colitis model increased. However, the spleen index (spleen weight/weight (g)) was restored in a dose-dependent manner in the OJE-treated group (Figure 3D). Furthermore, DSS-induced colonic shortening improved manner upon OJE treatment (100 or 200 mg/kg) (Figure 3E).

### 3.4. OJE Supplementation Reduced Expression of Pro-Inflammatory Cytokines and Activation of the NF-κB Signaling Pathway in Mice with DSS-Induced Colitis

**Figure 4:**
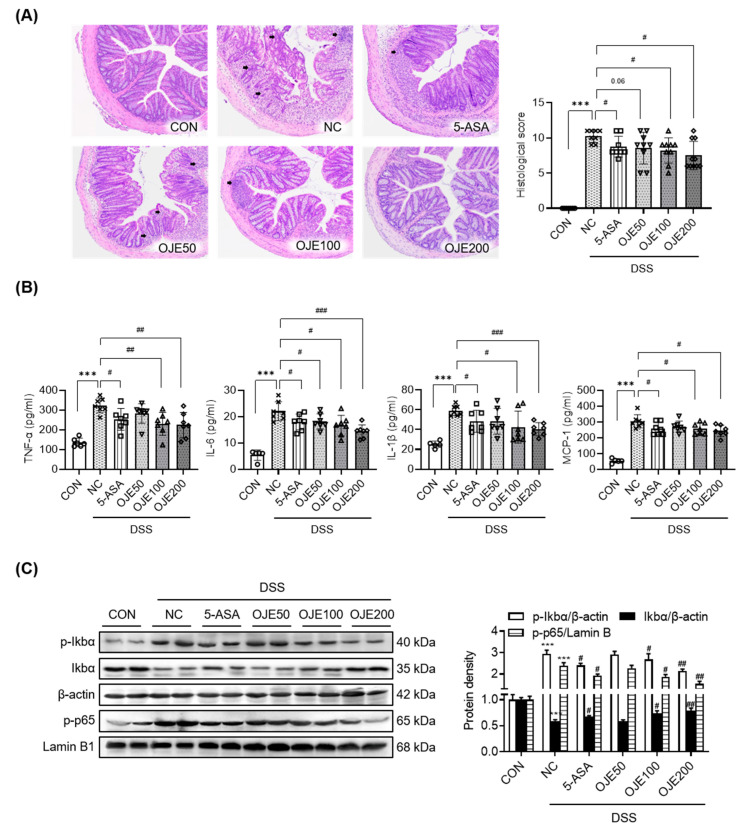
Effects of OJE on the histological change, serum levels, and activation of the NF-kB signaling pathway in mice with DSS-induced colitis. (**A**) H&E-stained colon sections (magnification 400×), histological score (*n* = 7–10), and (**B**) serum TNF-α, IL-6, IL-1β and MCP-1 levels as pro-inflammatory cytokines (*n* = 6–7). (**C**) Protein levels of total- and phosphorylated-IkBa and -p65 were analyzed via Western blotting. The two lanes show the bands tested in tissue extracts from two representative animals. Protein density was quantified (*n* = 6). Values are the mean ± SEM. *** *p* < 0.001, NC vs. CON; # *p* < 0.05, ## *p* < 0.01, and ### *p* < 0.001, 5-ASA 50 mg/kg, OJE 50 mg/kg, OJE 100 mg/kg, OJE 200 mg/kg vs. DSS+NC.

Histopathological changes in colon tissues were investigated using H&E staining. The pathological findings showed that immune cell infiltration into the distal mucosa of the colonic mucosa and epithelial cell destruction of the colon tissue significantly (*p* < 0.001) increased in the DSS group compared to those in the control mice (Figure 4A). However, administration of OJE significantly (*p* < 0.05) restored colon tissues in a dose-dependent manner.

Consistent with these histological changes, OJE treatment (100 or 200 mg/kg) significantly (*p* < 0.05) inhibited the serum levels of cytokines/chemokines (Figure 4B). Next, we evaluated the effect of OJE on the NF-κB signaling pathway, as NF-κB is an important transcriptional regulator of genes involved in colonic inflammation and cytokine expression. OJE administration significantly (*p* < 0.05) reduced tissue levels of p-IκBα, IκBα, and p-p65 in DSS mice (Figure 4C).

### 3.5. OJE Supplementation Ameliorates Inflammation in Mice with DSS-Induced Colitis

**Figure 5:**
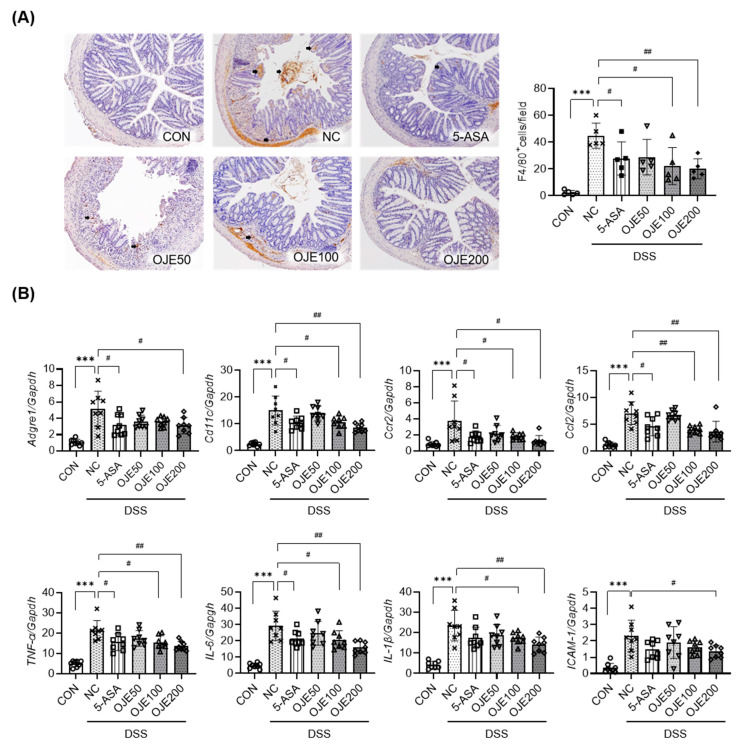
Effects of OJE on inflammation and oxidative stress in mice with DSS-induced colitis. (**A**) After 14 d of DSS treatment, colon tissues were retrieved and subjected to F4/80 immunostaining. F4/80-positive cells were counted (the bar graph shows quantified data derived from five representative animals, *n* = 5). (**B**) mRNAs of genes related to inflammation in colon tissues were analyzed using qPCR (*n* = 8). Values are the mean ± SEM. *** *p* < 0.001, NC vs. CON; # *p* < 0.05, and ## *p* < 0.01, 5-ASA 50 mg/kg, OJE 50 mg/kg, OJE 100 mg/kg, OJE 200 mg/kg vs. DSS+NC.

Next, we evaluated the role of OJE in colonic inflammation in mice with DSS-induced colitis. To assess macrophage infiltration, we immunostained the colon tissue sections with anti-F4/80 antibodies. Mice with DSS-induced colitis had a markedly higher number of F4/80-positive cells that accumulated in the colon tissues (Figure 5A). qPCR analyses also indicated severe colon tissue inflammation in mice with DSS-induced colitis, with a marked increase in mRNA levels of macrophage-related genes (F4/80 encoded by *Adgre1* and *CD11c*), chemokine *CCL2* and its receptor (*CCR2*), various cytokines (*TNF-α, IL-6,* and *IL-1β*), adhesion molecule *ICAM-1*, and serum levels of *TNF-α, IL-6, MCP-1,* and *IL-1β* (Figure 5B). Treatment with OJE caused a significant (*p* < 0.05) reduction in the amount of colonic macrophage infiltration and associated inflammatory markers compared to DSS control mice. These results suggested that OJE suppresses the early stages of colonic tissue damage by decreasing macrophage infiltration and the subsequent cytokine/chemokine expression.

### 3.6. OJE Supplementation Increased Expression of phase 2 Antioxidant Enzymes in Mice with DSS-Induced Colitis

**Figure 6:**
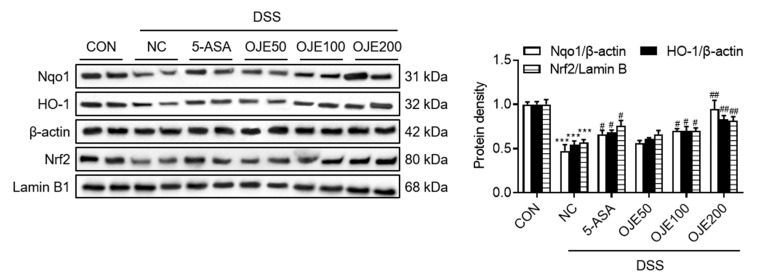
Effects of OJE on phase 2 antioxidant enzymes in mice with DSS-induced colitis. Protein expression of Nrf2, HO-1, and NQO1 in colon tissue. Values are the mean ± SEM (*n* = 6). *** *p* < 0.001, NC vs. CON; # *p* < 0.05, ## and *p* < 0.01, 5-ASA 50 mg/kg, OJE 50 mg/kg, OJE 100 mg/kg, OJE 200 mg/kg vs. DSS+NC.

As numerous antioxidant enzymes are regulated by the transcription factor nuclear factor erythroid 2-related factor 2 (Nrf2) [38], we examined whether OJE ameliorates colitis by activating the Nrf2 pathway. To confirm the effect of OJE administration on DSS-induced oxidative stress, relevant phase 2 antioxidant enzymes were quantified. As observed, the expression of NQO1, HO-1, and Nrf2 which are well known important factors of oxidative stress, significantly (*p* < 0.001) decreased in the NC group compared to the normal group. However, administration of OJE significantly (*p* < 0.05) increased the expression of NQO1, HO-1, and Nrf2 compared to that in the DSS group (Figure 6).

### 3.7. Changes in Gut Microbiota of Mice with DSS-Induced Colitis after OJE Supplementation

**Figure 7:**
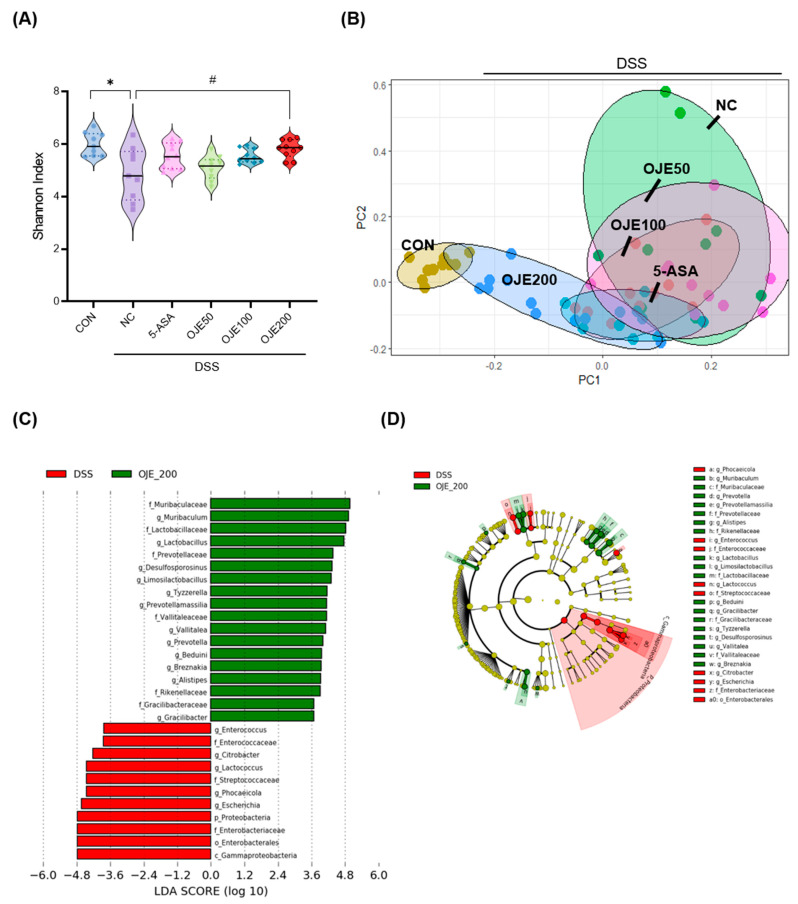
Differences in fecal microbiota with OJE administration on mice with DSS-colitis. (**A**) Alpha diversity analysis index (vertical lines represent medians in group), (**B**) Weighted unifrac plot, (**C**) LDA effect size plot, (**D**) Cladogram plot. Values are the mean ± SEM (*n* = 8–10). * *p* < 0.05, NC vs. CON; # *p* < 0.05, 5-ASA 50 mg/kg, OJE 50 mg/kg, OJE 100 mg/kg, OJE 200 mg/kg vs. DSS+NC.

The α-diversity was calculated using the Shannon Index to estimate the ability of OJE treatment to restore the gut microbial diversity (Figure 7A). The Shannon Index was significantly (*p* < 0.05) lower in the NC group than in the CON group. The group treated with 200 mg/kg OJE showed a significant (*p* = 0.02) increase in the Shannon Index compared to the NC group, and no significant difference was found in the 5-ASA group. However, low concentrations (50 and 100 mg/kg) of OJE were not significantly different from NC. β-Diversity is an index that indicates individual similarities based on the type and composition ratio of microbes. Figure 7B shows the PCoA results based on the distance values among groups. Together with the 5-ASA group, the OJE group showed a concentration-dependent shorter distance to the CON group, and the distance to the NC group tended to increase. Notably, the OJE-200 group had the shortest distance from the CON group. LEfSe is a tool to find significantly different microbes between two groups using relative abundances. The log10 transformed Linear Discriminant Analysis (LDA) represents the effect size for particular taxa in two groups. LEfSe showed a significant increase in the genus *Muribaculum* and a significant (LDA >4.0, *p* < 0.05) decrease in the phylum *Proteobacteria* in the OJE-200 group compared to the NC group (Figure 7C,D). At the family level, a greater abundance of *Muribaculaceae* and *Prevotellaceae* bacteria and a lower abundance of *Enterobacteriaceae* and *Streptococcaceae* were found in the OJE-200 group than in the NC group.

## 4. Discussion

IBD is a chronic recurrent bowel inflammatory disease, and its incidence is increasing in Western countries, Republic of Korea, and other Asian countries. Despite clinical treatment of IBD with glucocorticoids and aminosalicylate, some side effects (sickness, epigastric discomfort, and headache) have occurred, and medical costs for patients have increased because long-term administration is recommended. To reduce side effects and develop an effective treatment for IBD, processed phytochemicals extracted from edible plants that have been ingested for ages are used. *O. javanica* (Blume) DC (water parsley) has been steadily consumed as an edible vegetable; therefore, it is expected to be safe for the human body and minimizes side effects as a raw material for treating diseases. It has also been used as a traditional anti-inflammatory agent in Asia, and contains phenolic compounds. Thus, we hypothesized that phenolic compounds in water parsley could help improve inflammation, such as colitis.

The pathological hallmark of UC is an excessive inflammatory response in the intestinal mucosa [39]. We investigated whether the production of iNOS and COX-2, which induce pro-inflammatory cytokine production, and pro-inflammatory cytokines TNF-α and IL-1β could be inhibited by OJE administration in LPS-stimulated Raw 264.7 cells. We also confirmed that severe damage to the intestinal barrier was effectively ameliorated by OJE administration in mice with DSS-induced colitis. The expression of NF-κB and various cytokines was investigated to determine the effect of OJE on the inflammatory response. The transcription factor NF-κB induces the expression of a variety of pro-inflammatory genes, including those encoding cytokines and chemokines. UC, an inflammatory disease, significantly increases NF-κB expression, resulting in the increased production of various cytokines by iNOS and COX-2, which mediate inflammatory responses [40]. We confirmed that OJE administration inhibited IκB phosphorylation, prevented the dissociation of NF-κB from complexes with IκB, and prevented NF-κB from entering the nucleus. It also inhibits the production of pro-inflammatory cytokines IL-6, IL-1β, and TNF-α by producing iNOS and COX-2 enzymes. Moreover, it inhibits MCP-1, which plays an important role in macrophage/monocyte activity.

Pro-inflammatory cytokines have been reported to be key factors in the pathophysiology of IBD, which can induce intestinal and mucosal inflammation [41]. Our data showed that OJE administration reduced the levels of pro-inflammatory cytokines and related markers in mice with DSS-induced colitis.

The number of F4/80-positive macrophages present in the colon tissue was significantly (*p* < 0.05) inhibited by OJE administration. In addition, the result of confirming the gene expression of colonic inflammatory mediators suggested that macrophage infiltration into the damaged site is suppressed by downregulating the expression of the macrophage chemokine CCL2 and the adhesion molecule ICAM-1. The transcription of proinflammatory mediators (cytokines and chemokines) and adhesion molecules is regulated, in part, by the transcription factor NF-kB [42]. Macrophage-specific NF-kB dynamics can isolate patients with IBD [43], and inhibitors, such as ASA and sulfasalazine, in models of DSS-induced colitis have been shown to reduce NF-kB activity and macrophage penetration [44]. Consistent with this finding, OJE supplementation reduced NF-kB expression. Phosphorylation in colon tissue in DSS mice corresponds to lower levels of cytokines in the serum and less macrophage infiltration in colon tissue.

Excessive damage to the immune defense system of the gut has been implicated in IBD pathogenesis. Pro-inflammatory cytokines that promote the production of reactive oxygen species (ROS) in immune cells are known to induce oxidative stress. Continuous stimulation of oxidative stress caused by ROS destroys genes and reduces the ability of cells to regenerate, eventually weakening the immune system and leading to diseases, such as inflammatory colitis [45]. The Nrf2 pathway, which is activated by ROS, plays an important role in protecting gut integrity through the regulation of antioxidant enzymes and pro-inflammatory cytokines produced by oxidative stress in UC. When Nrf2 is exposed to oxidative stress, it translocates from the cytoplasm to the nucleus, inducing the expression of various antioxidant genes such as HO-1 and NQO1 to protect cells [46]. Therefore, we investigated the protein expression of Nrf2, HO-1, and NQO1 to determine whether OJE administration affects oxidative stress in mice with DSS-induced colitis. The protein expression of Nrf2, HO-1, and NQO1, which are phase 2 antioxidant enzymes, significantly (*p* < 0.001) decreased in DSS-induced colitis, and was significantly (*p* < 0.05) increased by OJE administration.

Among the factors influencing the pathogenesis of IBD, the importance of gut microbiota has increased with further experimentation. More than 200 risk factors are associated with IBD in the human genome, and several are related to interactions between gut microbiota [47]. In general, the intestinal microbiome of patients with IBD shows an increase in facultative anaerobic bacteria and decrease in obligatory anaerobic bacteria, which are both closely related to short chain fatty acids (SCFAs) production [48,49]. A decrease in the phylotype richness and evenness (alpha diversity) of the microbiota is a typical phenomenon in several diseases, including IBD [50]. In this study, the administration of 200 mg/kg OJE resulted in significant increases in alpha diversity, such as the Shannon Index. It has been suggested that the ingestion of OJE into the host via oral administration can increase the types and balance of intestinal microbes that have been reduced because of inflammation, and restore a healthy state.

Notably, a decrease in *Firmicutes* and a rapid increase in *Proteobacteria* among the gut microbiota are typically found in IBD [51]. In this study, we confirmed that the administration of OJE drastically reduced the abundance of *Proteobacteria* and that of the genus *Escherichia*. It is unclear whether OJE administration suppresses the growth of pathogenic bacteria or whether it promotes the growth of competitors, causing a relative decline; however, these results can be considered another indicator of colitis improvement. *Muribaculum*, commensal gut bacteria, have been demonstrated in several studies to be the dominant bacterial group in healthy mice [52]. Oral administration of OJE maintained a high abundance of *Muribaculum*. This means that, together with the reduction of *Proteobacteria*, inflammatory bacteria, OJE treatment maintains or recovers a healthy state. However, it is difficult to identify clear contributing pathways to the amelioration of colitis because of the lack of insight into the functional potential of *Muribaculum*.

Phenols are a large family of compounds containing a hydroxyl group attached to an aromatic hydrocarbon group. Abundant evidence for the potent anti-inflammatory and antioxidant activity of phenolic acids in vitro and in vivo [53] suggests their potential for preventive and therapeutic effects on IBD. Phenolic compounds are primarily detected in plants with green leaves, and *O*. *javanica* contains abundant and varying phenolic acids. Apigenin [54], quercetin [55], isorhamnetin-3-O-galactoside [23], afzelin [56], luteolin, kaempferol, and rutin [57] are the major phenolic compounds in *O. javanica*. Therefore, we hypothesized that the phenolic compounds in OJE are closely related to the effect of relieving inflammation and inducing changes in the intestinal microbiota. We identified three phenolic compounds in OJE, which were NO inhibitors associated with anti-inflammatory properties [58]. Among the main components, chlorogenic acid has been shown to protect against inflammation and oxidative stress in obese mice, while simultaneously improving the gut microbiome [59]. In addition, ferulic acid exhibited antioxidant and anti-inflammatory properties in transverse aortic constriction (TAC) mice, a model of heart disease, and increased the intestinal *Lactobacillus* effect, eventually suppressing heart disease [60]. Rutin (quercetin-3-rutinoside) is also known to act as an antioxidant [61], and the results of this study and other studies suggest that all three key indicators potentially act directly or indirectly in relieving colitis.

## 5. Conclusions

This study has several limitations. First, phenolic components present as the main ingredients of OJE in cells or animals have not been individually investigated for cytokine production associated with inflammatory responses. Second, through LC/MS analysis of OJE, other peak components could not be investigated, except for three substances that were selected as the main components. Finally, metabolite profiling of the blood or feces to confirm changes in intestinal microbial metabolism due to OJE administration was not performed.

In conclusion, we demonstrated the anti-inflammatory and anti-oxidant activity and the regulation of gut microbiota composition beneficial effects of OJE in LPS-induced macrophages and DSS-induced colitis mouse models. In particular, the anti-colitis effect of OJE (200 mg/kg) was similar to or better than that of 5-ASA (50 mg/kg) in most factors. OJE affects the intestinal microbiome ecosystem in colitis and significantly increases beta diversity in colitis mice. This is indicative of reduced gut microbial diversity and differences in the gut microbiota type and abundance between individuals. It has also been shown to restore intestinal microbial communities close to those in healthy mice. There was no significant difference between high-dose OJE and the currently used colitis treatment, 5-ASA. Considering that the OJE used in this study is not a single ingredient, and a natural plant extract containing various ingredients, the fact that it has a similar effect to 5-ASA means that it has great potential for practical use. Therefore, we propose that OJE can be applied as a potential medicinal plant for the amelioration and prevention of colitis, an inflammatory disease.

We propose that further in vivo investigations are required to understand the exact mechanisms and anti-inflammatory effects of OJE phenolic components on the relationship between digestive processes and gut microbiota. Moreover, with further studies, metabolite profiling is necessary to establish a clear correlation between metabolites mediated by the gut microbiota following OJE administration.

## Data Availability

The data is contained within the article and the supplementary material.

## Supplementary Materials

The following supporting information can be downloaded at: https://www.mdpi.com/article/10.3390/antiox11122429/s1, Table S1: Sequences and accession numbers for primers (forward, FOR; reverse, REV) used in real-time RT-PCR; Table S2: Mass fragmentation ions of 3 components from OJE.; Table S3: Histological scoring of colitis.
