# Supplementary material for: Oenanthe javanica Ethanolic Extract Alleviates Inflammation and Modifies Gut Microbiota in Mice with DSS-Induced Colitis
Source: Antioxidants (Basel). 2022 Dec 9;11(12):2429. doi: 10.3390/antiox11122429 (PMC9774932; doi:10.3390/antiox11122429)
Supplement: Supplementary file 1 [file antioxidants-11-02429-s001.zip › antioxidants-2058824-supplementary.pdf]

Table S1. Sequences and accession numbers for primers (forward, FOR; reverse, REV) used in real-time RT-PCR

Gene	Sequences for primers	Accession No.
iNOS	FOR: CTCACTGGGACAGCACAGAA	NM_010927
	REV: GGTCAAACTCTTGGGGTTCA	
COX-2	FOR: TACAGAATTGAAAGCCCTCT	NM_011198
	REV: GCTCGGCTTCCAGTATTGAG	
TNF-α	FOR: AGGGTCTGGGCCATAGAACT	NM_013693
	REV: CCACCACGCTCTTCTGTCTAC	
IL-1β	FOR: GGTCAAAGGTTTGGAAGCAG	NM_008361
	REV: TGTGAAATGCCACCTTTTGA	
IL-6	FOR: AGGGTCTGGGCCATAGAACT	NM_031168
	REV: CCACCACGCTCTTCTGTCTAC	
F4/80 (Adgre1)	FOR: TTTCCTCGCCTGCTTCTTC	NM_010130
	REV: CCCCGTCTCTGTATTCAACC	
CD11/c	FOR: CACTCAGTGACTGCCCAAAA	NM_021334
	REV: CCTCAAGACAGGACATCGCT	
CCR2	FOR: AGCACATGTGGTGAATCCAA	NM_009915
	REV: TGCCATCATAAAGGAGCCA	
CCL2(MCP-1)	FOR: ATTGGGATCATCTTGCTGGT	NM_011333
	REV: CCTGCTGTTCACAGTTGCC	
ICAM-1	FOR: AACAGTTCACCTGCACGGAC	NM_010493
	REV: GTCACCGTTGTGATCCCTG	
GAPDH	FOR: CGTCCCGTAGACAAAATGGT	NM_008084
	REV: TTGATGGCAACAATCTCCAC	

Table S2. Mass fragmentation ions of 3 components from OJE.

Peak	Component	MW[1)]	Rt(min)[2)]	Fragment ions
1	chlorogenic acid	354	8.853	377.0844 $[M+Na]^+$,<br>355.1025 $[M+H]^+$,<br>169.0389 $[Caffeoyl]^+$
2	5-*O*-feruloylquinic acid	368	12.978	391.1004 $[M+Na]^+$,<br>369.1180 $[M+H]^+$,<br>177.0546 $[Fe^{3)}+H-H_2O]^+$,<br>145.0285 $[Fe+H-H_2O-CO-CH_3]^+$,<br>117.0336 $[Fe+H-H_2O-CH_3OH-CO]^+$
3	rutin<br>(quercetin-3-rutinoside)	610	16.834	633.1433 $[M+Na]^+$<br>611.1612 $[M+H]^+$<br>465.1033 $[M+H-Rhamnose]^+$<br>303.0502 $[Quercetin aglycone+H]^+$

[1)]Molecular weight. [2)]Retention time. [3)]Ferulic acid

Table S3. Histological scoring of colitis

Parameter	Scoring
Crypt loss	0=normal<br>1=<10%<br>2=10%<br>3=10-15%<br>4=15-50%
Erosions	0=intact epithelium<br>1=involvement of the lamina propria<br>2=involvement of the submucosa<br>3=transmural ulceration
Infiltration of mononuclear and polymorphonuclear cells	0=absent<br>1=weak<br>2=moderate<br>3=severe
